# Biosecurity aspects of cattle production in Western Uganda, and associations with seroprevalence of brucellosis, salmonellosis and bovine viral diarrhoea

**DOI:** 10.1186/s12917-017-1306-y

**Published:** 2017-12-06

**Authors:** C. Wolff, S. Boqvist, K. Ståhl, C. Masembe, S. Sternberg-Lewerin

**Affiliations:** 1 Department of Biomedical Sciences and Veterinary Public Health, Swedish University of Agricultural Sciences, Uppsala, Sweden; 2 Department of Disease Control and Epidemiology, National Veterinary Institute, Uppsala, Sweden; 3 College of Natural Sciences, Makerere University, Kampala, Uganda

**Keywords:** Serology, Biosecurity, Poisson regression, Additive bayesian network, Endemic infections, Disease control, Uganda, Cattle

## Abstract

**Background:**

Many low-income countries have a human population with a high number of cattle owners depending on their livestock for food and income. Infectious diseases threaten the health and production of cattle, affecting both the farmers and their families as well as other actors in often informal value chains. Many infectious diseases can be prevented by good biosecurity. The objectives of this study were to describe herd management and biosecurity routines with potential impact on the prevalence of infectious diseases, and to estimate the burden of infectious diseases in Ugandan cattle herds, using the seroprevalence of three model infections.

**Results:**

Farmer interviews (*n* = 144) showed that biosecurity measures are rarely practised. Visitors’ hand-wash was used by 14%, cleaning of boots or feet by 4 and 79% put new cattle directly into the herd. During the 12 months preceding the interviews, 51% of farmers had cattle that died and 31% had noticed abortions among their cows. Interestingly, 72% were satisfied with the health status of their cattle during the same time period. The prevalence (95% CI) of farms with at least one seropositive animal was 16.7% (11.0;23.8), 23.6% (16.9;31.4), and 53.4% (45.0;61.8) for brucella, salmonella and BVD, respectively.

A poisson regression model suggested that having employees looking after the cattle, sharing pasture with other herds, and a higher number of dead cattle were associated with a herd being positive to an increasing number of the diseases. An additive bayesian network model with biosecurity variables and a variable for the number of diseases the herd was positive to resulted in three separate directed acyclic graphs which illustrate how herd characteristics can be grouped together. This model associated the smallest herd size with herds positive to a decreasing number of diseases and having fewer employees.

**Conclusion:**

There is potential for improvement of biosecurity practices in Ugandan cattle production. Salmonella, brucella and BVD were prevalent in cattle herds in the study area and these infections are, to some extent, associated with farm management practices.

**Electronic supplementary material:**

The online version of this article (10.1186/s12917-017-1306-y) contains supplementary material, which is available to authorized users.

## Background

In low-income countries cattle represent a source for nutritious food, income, and may also play a major role in the social context [1]. One threat to livestock production is infectious diseases, endemic or epidemic, which directly affect the farmer but also many formal and informal actors in the often complex value chains of animal products [2]. Outbreaks of infectious, serious and so called Transboundary Animal Diseases (TADs) in cattle may have additional consequences for the farmers due to trade restrictions. Such diseases, and others with high morbidity and clear clinical signs and/or increased mortality are likely to be recognised by farmers, and linked to losses in production [3]. However, endemic infections with less dramatic clinical manifestations, such as reproductive disturbances, diarrhoea, or poor growth, may be part of what farmers perceive as “normal” and not as something possible and/or worthwhile to control, despite negative effects on income and food security.

The prospects for eradication of specific infectious diseases might be beyond what is reasonable in low-income countries, where the livestock production systems differ from systems in high-income countries. Instead, efforts could be made to decrease infection pressure in general, by improved biosecurity, and thus improve productivity.

Biosecurity, i.e. actions to prevent introduction of infectious agents in an area, farm or group of animals, or to limit circulation of those already present, is essential for animal disease control and improved herd health [4]. Many biosecurity measures are not disease-specific, e.g. to keep animals separated from other herds. To successfully suggest changes in biosecurity to cattle farmers one has to know what management routines are currently practiced in the population. In addition, knowledge of actual associations between disease occurrence and management in the local context would give valuable information on current routines with high impact on disease prevalence in cattle.

Uganda is an East African country with great potential for food production and for developing its livestock sector. The bovine disease spectrum is similar to other Sub-Saharan African countries and, in contrast to countries with a more developed dairy production, dominated by infectious diseases. Farmers themselves rank east coast fever (ECF), helminthosis, trypanosomiasis, anaplasmosis, and foot-and-mouth disease (FMD) as major disease problems [5]. As many infectious diseases in cattle are zoonotic and of a public health concern, control of such diseases in cattle is not only an economic issue, but a One Health issue. When disease control actions are carried out, these generally focus on response to outbreaks, e.g. vaccination and movement bans during FMD outbreaks [6]. Research efforts have been targeted mainly at TADs, such as FMD and vector-borne diseases, e.g. trypanosomiasis [7–9]. The knowledge regarding many other endemic and less obvious infectious diseases that are likely to have a high impact on calf mortality, reproduction, growth and milk production, is more limited. Estimating the occurrence of such diseases may provide a better picture of the overall disease burden of infectious diseases in the cattle population. Further, if and to what extent Ugandan cattle farmers practice biosecurity is unknown. This knowledge would be useful for future interventions.

This study is part of a project on biosecurity as a tool for cattle disease prevention. The objectives were: firstly, to describe herd management and biosecurity routines with potential impact on the prevalence of infectious diseases in Ugandan cattle herds and, secondly, to estimate the burden of infectious diseases and any associations with herd management, using three model infections.

## Methods

This was a cross-sectional field study with the herd as the unit of interest.

### Cattle production in Uganda

Cattle are mainly kept in extensive pasture-based production systems: tethered, in enclosures or with communal grazing. At the last livestock census the country had a population of 11.4 million cattle heads and about 1 in 4 households keep cattle. An estimated 1.5 million cows were milked for household consumption and/or for family income. The average milk production per cow and week was 8.5 l [10]. The low production can partly be attributed to the use of local breeds or local breeds crossed with exotic (mainly European) breeds, poor or sub-optimal feeding, a lack of breeding programmes, general lack of production planning and a high disease burden [1, 11]. People and animals live closely together. Meat and dairy value chains are characterised by short distances to the end consumer, promoting spread of zoonotic infections between cattle and humans via milk, water, soil or direct contact [12, 13].

Extension services for farmers are lacking in capacity in Uganda due to a shortage of qualified veterinarians [14]. Farmers can buy antibiotics, anti-parasitics, tick-treatment or prophylactics over-the-counter in “veterinary shops” where the staff is not required to have any education in veterinary medicine. Moreover, farmers can often not afford veterinary services and wait until they have tried various treatments including medical drugs or traditional remedies before calling a veterinarian [15, 16]. In each district a District Veterinary Officer (DVO) is the governmental representative responsible for animal health and disease control, surveillance and reporting to the Ministry of Agriculture, Animal Industry and Fisheries. The DVOs can have one or several Veterinary officers and paraveterinarians employed in their unit. Veterinary officers and veterinary assistants/paraveterinarians can also be self-employed but contracted by the DVO for e.g. government vaccination campaigns or externally funded projects.

### Selection of study herds

**Fig. 1:**
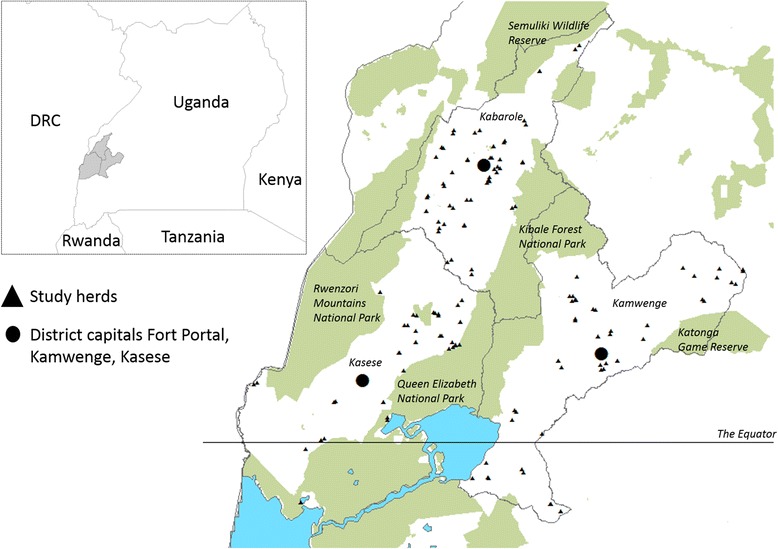
Map of the study area in Western Uganda with districts Kabarole, Kamwenge and Kasese

The study area was the neighbouring districts Kabarole, Kamwenge and Kasese in South-Western Uganda, an important area for livestock production (Fig. 1). These districts were chosen because studies on bovine infectious disease are sparse from this specific area and together they include several agro-ecological zones with expected varying herd sizes and management systems (although no detailed data were available). Each district is organised in administrative units of sub-counties, further divided in parishes (or wards in cities) that are subsequently divided in villages (or zones).

At the livestock census in 2008 [10] the estimated number of cattle per district was 67,120, 120,190 and 97,240 in Kabarole, Kamwenge and Kasese, respectively. There were 15,530, 14,100, and 5530 households with at least one bovine animal and the mean (median) herd sizes were 4.3 (3) in Kabarole, 1.8 (8.6) in Kamwenge and 17.6 (11) in Kasese [10]. The DVOs provided estimates of what they perceived as the current distribution of herd sizes in their districts. These herd sizes were larger than in the 2008 census.

To estimate the sample size needed to assess the prevalence of the model diseases at herd level (positive or negative herd) the online tool Epitools [17] was applied. Confidence levels of 95%, 5 to 10% precision, herd sensitivity and specificity in the range of 0.7 to 0.99 and true prevalences from 1 to 50% were evaluated. A total sample size of 180 herds, i.e. 60 per district, was decided upon based on what would be practically possible to manage and allow prevalence estimates of reasonable precision.

A list of all administrative units in Uganda was entered into a Microsoft® Excel (Microsoft Corporation, Redmond, USA) spreadsheet and a simple random selection, using the random number function, of 30 villages was made in each of Kabarole and Kamwenge districts. In Kasese, three quarters of the villages in the initial random sample had, according to the DVO, no cattle because they were located in mountains or in a national park. To reach the desired number of villages, a purposive sample, geographically spread over the district, was made from the list of the remaining villages which the DVO confirmed had at least two cattle herds.

Next, each selected village was visited by a local veterinary officer or veterinary assistant. In Kabarole and Kasese, the village chairperson was asked to list all farmers owning at least two cattle. On a second visit a simple random sample of two herds was made from this list. The selection was made by writing each farmer’s name on a piece of paper which was folded and put into a container. The selected farmers were asked if they agreed to participate in the study. If consent was not given, another farmer was selected by the same method. In Kamwenge district, selection of the first farmers was made by driving 5 min east from the village centre and asking the nearest farmer with at least two cattle if (s)he was willing to participate, if not, the next farmer in that direction was asked until two farmers were recruited. This procedure was chosen because of few local team members and poor road conditions.

### Data collection

Herds were visited for sampling during January to March 2015 by the first author and a team of 2-5 local veterinary officers and veterinary assistants. The local teams were informed about the purpose of the study before recruiting farmers, and before the data collection visit they were further trained for the interviews, on information to be provided to farmers and biosecurity measures for the team.

In total 144 herds were visited and sampled: 55, 49, and 40 in Kabarole, Kamwenge, and Kasese, respectively. One farm was included because the owner was a prominent member of society and the herd vet, i.e. a local team member, requested that the herd should be part of the project. Six herds were added on the day of sampling; when the originally planned visits could not be carried out the first author asked the local team if they knew any farmers in the village or along the way back to the district headquarters that might be willing to participate. Three of these additional herds had owners that were prominent members of society.

A two-page questionnaire was used for the farmer interviews. The questionnaire was pilot-tested with the DVOs and their assistants before the study, and some questions were revised for improved clarity. The 39 questions were mainly closed (categorical or quantitative) or semi-closed. The topics covered included herd management, reproductive performance, cattle trade, animal mortality, vaccination, and disease prevention measures carried out. The questionnaire (in English) is available as additional file 1.

During the farm visit, a local assistant interviewed the farmer in any of the local languages (Rutoro, Rukhiiga or Rukonzo) with the first author observing and asking for clarification when needed, or the first author asked the questions in English and the local assistant translated. When possible, the answers to the interview questions were verified, e.g. herd size or the presence of other livestock species.

Interview data were coded and entered into a Microsoft® Access database (Microsoft Corporation, Redmond, USA) where possible with restrictions on values valid for entry. After data entry, each interview record was manually checked for data entering errors.

The coordinates of each farm were collected during the farm visit using the GPS tracker app My Tracks on an android mobile device. A map of the study area with the location of all study herds indicated was created in ArcMap 10.3.1 (Esri, Redlands, USA) using shape files available via ArcGis Online and from Free Spatial Data (DIVA-GIS).

### Model diseases

Three infections were chosen as model diseases: brucellosis, salmonellosis and bovine viral diarrhoea (BVD). The choice of model diseases was based on expected endemicity in the study area, and availability of commercial serological assays that could be run in Uganda. The diseases have different transmission routes, together representing those of many other infections. They also differ in contagiousness but can be prevented by good biosecurity. The clinical manifestations include reproductive disorders such as abortions, infertility and prolonged calving intervals (brucella, salmonella, BVD), calf morbidity and mortality due to respiratory illness and diarrhoea (salmonella, BVD), loss in milk yield (brucella, salmonella, BVD), lameness and swelling of joints (brucella). Infections can be clinically manifest or subclinical. Salmonellosis and brucellosis are also important zoonoses [18, 19]. When introduced in a herd, infection can persist in individual animals and/or remain in the herd if new susceptible animals are added. Animals that have been infected with either of these agents remain seropositive for years (brucella and BVD) or months (salmonella).

The seroprevalence of brucellosis in Ugandan cattle has been estimated to range from 6.5% on herd level and 5.0% on animal level [20] to 55.6% at herd level and 15.8% at animal level [21], and varying from 0 to 78% at herd level depending on year and area [22]. No reports from the current study area could be found but in two neighbouring districts the animal prevalence was estimated to 14% [23]. However, Kabi et al. [24] reported that the seroprevalence in Ugandan cattle varies between agro-ecological zones. The within-herd prevalence of bovine brucellosis has been estimated to 26% in peri-urban and urban Kampala [20] and from 1 to 90% in Mbarara district [21].

For salmonella and BVDV, peer reviewed reports of seroprevalence in Ugandan or African cattle appear sparse. However, an MSc study suggested a seroprevalence at herd level of 57 and of 24% on individual level for salmonella, and 39% at herd level and 23% on individual level for BVD, in urban and peri-urban Kampala [25]. The seroprevalence of BVD has been reported as 92% at herd level in Cameroon [26], and 12% at the individual level in Tanzania [27].

### Sample collection

Epitools was used to calculate the number of animals to be tested in a herd to detect at least one positive animal [28]. Based on the herd size information from the DVOs, herd sizes from 10 to 100 animals were evaluated with a confidence level of 95%, desired herd sensitivity of 95%, and test sensitivity of 98%. The final sample size was as follows: in herds with up to 20 cattle, all animals were sampled; in herds with 21 to 50 cattle, 20 animals were sampled; and in herds with more than 50 cattle, 30 animals were sampled.

Where possible, and based on intra-herd epidemiology of the respective diseases and expected seroconversion, animals between approximately 5 months and 2 years of age were prioritised for blood sampling, to get the recent infection history of the herd. Such animals are hereafter referred to as “young” individuals and older animals as “adult”. The age group was determined based on the owners’ information and the local teams’ judgement, but age could not be ascertained in more detail.

Four farms had only one animal on the day of the herd visit because one animal had been sold or had died since recruitment. For 17 herds, fewer cattle than planned were bled for reasons including: cattle escaped, were too aggressive to handle, only calves were available, or because the farmer was unable to assist the team.

Blood was collected with Vacutainer® from the jugular or coccygeal vein into sterile tubes with no additive. Samples were stored in a cool box during transport to the field laboratory where they were centrifuged at 3000 rpm for 15 min and sera transferred to Greiner cryo vials. Within 2 days the refrigerated sera were transferred to a deep freezer (exact temperature varied but always below the freezing point) until transported to the laboratory at Makerere University in Kampala. At the laboratory, samples were stored in −20° C or −80° C until analysed.

All handling of animals including sampling was carried out under the direct supervision of the District Veterinary office staff in accordance with their national mandate.

### Serological assays

Serum samples were analysed at Makerere University in Kampala, using commercial ELISAs. The assays were run according to the manufacturers’ instructions. The person performing the analyses was blinded to information about the samples except the sampling date and district of origin.

For brucella, the SVANOVIR® Brucella-Ab I-ELISA (Svanova, Uppsala, Sweden) which detects antibodies to *B. abortus* and *B. melitensis* was used. According to the manufacturer, the test had a sensitivity (Se) of 100% in brucella-positive individuals and a specificity (Sp) of 100% in a brucella-negative population.

For salmonella, the PrioCHECK® Salmonella Ab bovine ELISA was used (Thermo Fischer Scientific, Waltham, Massachusetts, USA). It detects antibodies directed against *Salmonella* serotypes belonging to the B and D group, specifically the O-antigens: 1, 4, 5, 12 and 1, 9 and 12. The manufacturer has not provided the Se and Sp of this assay.

For BVD the SVANOVIR® BVDV-Ab (Svanova, Uppsala, Sweden) which detects both genotypes of BVD virus was used. According to the manufacturer the Se is 100% and the Sp 98.2% for serum samples.

For BVD it has been shown that it is sufficient to test a small number of individuals when the objective is to detect an ongoing or recent infection in the herd [29] and up to seven samples per herd were analysed, fewer for herds with <7 cattle. To save costs, serological analyses for brucella and salmonella were initially also performed on up to seven samples per herd. From herds where no positive samples were detected in this first round, more samples were analysed so as not to miss positive herds. For Brucella, all remaining samples from negative herds were analysed in a second round. For salmonella, up to nine more samples were analysed from the negative herds in a second round, while the remaining samples from herds that were still salmonella-negative were analysed in a third round. The number of samples included in each round of analysis was based on how many samples could be analysed with each ELISA kit. When possible, samples analysed first were from individuals of <2 years of age for salmonella and BVD to get the most recent infection history of the herd. For Brucella, individuals >2 years of age were prioritised because reproductively active (i.e. sexually mature) animals are at higher risk of clinical infection.

The manufacturers’ cut-off (percent positivity, PP) for positive test result was used as cut-off for the individual animals’ test result (PP 60 for brucella, 35 for salmonella and 10 for BVD). To check consistency and quality of the ELISA analyses, the distribution of PP values below the cut-off for a positive test, i.e. all negative individual samples, were compared between runs/plates (of the same ELISA) by visual inspection of plots of the distribution of values, by summary measures and by testing for difference with the Kruskal-Wallis test to ensure that negative samples were clearly below the cut-off and no individual plate/run deviated towards the cut-off-value.

### Data management and analysis

Data management and statistical analyses were performed in the statistical package R [30].

Descriptive statistics including tabulations, summary measures and plots of distributions of variables were produced at herd level and, for the serological data, also at the individual animal level.

Of the eight categories for herd size, the three categories for the largest herd sizes were merged to one (> 50 cattle) because of few observations. The five remaining categories reflected, roughly, different management systems. The dichotomous variables for use of own bull, communal bull, artificial insemination (AI) and “other” were combined to one categorical variable with the categories own bull, communal bull, and “other”. For seven variables there was very little variation, i.e. all responses were similar and therefore not suspected to explain any variation in herd serological status. These variables were not evaluated further. They included, for example, the use of tick spray for disease prevention, if cattle were kept on pasture, the number of cattle sold due to disease and the number of cattle returned from market unsold.

The remaining 29 variables were evaluated for associations between herd management and infectious disease burden. For each disease, a herd was defined as positive if at least one individual animal had a positive test result. A variable for the number of diseases a herd was positive to (0-3) was created. Apparent prevalence of positive herds for each disease with exact binomial confidence intervals were calculated in total, including only young or only adult individuals, and per district. Differences between districts were examined with Fisher’s exact test as were differences between districts for the number of diseases (0, 1, 2, or 3) a herd was positive to.

Differences between categories of categorical interview variables was assessed by Chi2 test or Fisher’s exact test and for numerical variables by the Kruskal Wallis test. All 29 variables were also evaluated in univariable poisson regression models for the outcome number of diseases a herd was positive to. If there was a difference with a *p*-value <0.2 the variable was evaluated further in the multivariable poisson regression model. Multicollinearity was evaluated by a Spearman rank correlation matrix including all the continuous variables, by a Spearman rank correlation matrix including all the binary variables, and for the categorical variables pairwise combinations were evaluated by the Chi-square test or Fisher exact test [31]. Where two variables were judged as correlated (correlation coefficient > 0.9 [31] or *p* < 0.05 in the Chi-square or Fisher exact test) the one which was highest in the hierarchy was selected for further evaluation, e.g. number of dead cattle above the number of dead calves and district above herd size. Herd size was correlated to several other variables but did not fulfil the criteria for a confounder as it was not on any causal pathway to the outcome. The initial multivariable poisson model included the binary (yes/no) variables for employees caring for the cattle, if cattle were tethered for pasture, if pasture was shared with other cattle herds, if cattle were watered from an open water source (e.g. a river), if there were pigs on the farm, and if the farmer was satisfied with the health status of the cattle in the previous 12 months. The initial model also included the categorical variables for bull and district, and the continuous variables for number of calves born, cattle that died, cattle that were sold or given away, and cattle bought or received, during the previous 12 months.

The model was reduced by removing the least significant variable one at a time and comparing it to the previous by a likelihood ratio test. The parameter estimates were checked for changes of >25% which would suggest the removed variable was a confounder and to be kept in the model. All removed variables were added to the final model one at a time to evaluate confounding. First order interactions between all main effects in the final model were tested. The final model was evaluated for overdispersion and residuals were visually examined. The number of observations included in the final model was 144.

Because of the many statistical tests carried out, a conservative significance level of *p* < 0.01 was applied when interpreting test results.

As the focus of our interest was biosecurity, to further explore the direct and indirect associations between herd routines that influence biosecurity and the serological status of herds, an Additive Bayesian Network (ABN) was fitted to the interview and serological data. An ABN is a multivariate model that describes the complex relationships of the direct and indirect connections between random variables. The ABN methodology is an extension of usual GLM models to include multiple response variables [32]. An ABN has two parts; the structure (qualitative) and the model parameters (quantitative). Each node comprises a generalized linear model where the arcs from parents of that node represent the covariates in the regression model. An ABN illustrates both direct and indirect associations between variables and each outcome variable [33]. A directed acyclic graph (DAG) graphically represents the joint probability distribution of all random variables in the data. The model parameters are represented by local probability distributions for all the variables in the network. A general introduction to ABN in veterinary epidemiology is given by Lewis et al. [33] and Lewis and Ward [34]. Other applications in veterinary epidemiology include associations between on-farm biosecurity practice and equine influenza [35], cattle herd risk factors and salmonella [36], and identification of *E. coli* antimicrobial resistance patterns [32].

Interview responses representing biosecurity measures, a variable classifying the herd as negative or positive to at least one of the diseases, herd size and district were included - in total 14 variables. The number of variables was reduced to adapt to the recommendation not to exceed 10% of the number of observations (personal communication M Pittavino, Institute of Mathematics, Applied Statistics Group, University of Zurich, Switzerland). As required for the analyses, multinomial variables were split into several binomial variables, and observations with missing data removed (*n* = 4). A hill climber search [37] with 1000 iterations was applied to identify a globally optimal consensus DAG for the data. The network score (the log marginal likelihood) was estimated using Laplace approximations at each node and used as the goodness of fit measure. The means and variances hyper parameters for the Gaussian priors were fixed at zero and 1000, respectively. The number of allowed parents per node, i.e. the number of covariates in the regression model of a node, was increased for each new search, starting at one, until the network score did not increase further. Arcs between binomial variables derived from the same multinomial variable, and arcs with the herd serology status as parent node were banned. The arcs in the consensus DAG were retained for the next search. The analysis was performed with the R library abn [38].

## Results

Herd sizes, production and management data from the farmer interviews are presented in Tables 1 and 2. As seen in the livestock census 2008 [14], small herds of less than five dominated in Kabarole district while Kasese district had the largest herds. The median number of animals per herd corresponded to those estimated in the census, except for Kasese where the median size in this study was 21–50 animals and the median figure reported in 2008 was 11 animals [10]. The most commonly stated main purpose of the cattle was dairy and beef (59.7%, *n* = 86), while dairy was the second most common (34.7%, *n* = 50). However, dairy was less common in Kasese (17.5%, *n* = 7) and the two farmers that stated beef production as the main purpose were in this district. Other purposes included e.g. draught power or not stated (*n* = 6). The most frequent breed was mixed breed (local and exotic) which was the main breed in 76 (53%) herds. The second most common breed was local breed, which was the main breed in 57 (40%) herds. Exotic breed was the most common breed in 11 (8%) herds. Most cattle were managed by the owner and his/her family but about a third of the cattle herds in Kabarole, half of those in Kamwenge and two thirds of those in Kasese also had employees taking care of the cattle. In Kabarole, most farmers (76.4%, *n* = 42) used a communal bull but ten farmers (18.2%) had their own bull and seven (12.7%) also stated that they use AI. Only one farmer in each of the districts Kamwenge and Kasese used AI and in Kamwenge about half of the farmers had their own bull and half used a communal bull. In Kasese, two thirds of the farms had their own bull and one third used a communal bull.

**Table 1:** Description of some of the non-numerical parameters regarding cattle herd management and performance in Western Uganda

	Kabarole *n* (%)	Kamwenge *n* (%)	Kasese *n* (%)	Total study area *n *(%)
Total	55	49	40	144
Herd size*				
2-5	35 (64)	13 (26.5)	2 (5)	50 (34.7)
6-10	11 (20)	14 (28.6)	7 (17.5)	32 (22.2)
11-20	4 (7.3)	11 (22.4)	6 (15)	21 (14.6)
21-50	5 (9.1)	6 (12.2)	14 (35)	25 (17.4)
51-100	0	3 (6.1)	9 (22.5)	12 (8.3)
101-150	0	0	0	0
151-200	0	2 (4.1)	2 (5)	4 (2.8)
Purpose of cattle *				
Beef	0	0	2 (5)	2 (1.4)
Dairy	24 (43.6)	19 (38.8)	7 (17.5)	50 (34.7)
Dairy and beef	29 (52.7)	30 (61.2)	27 (67.5)	86 (59.7)
Other	2 (3.6)	0	4 (10)	6 (4.2)
Cattle care by				
Husband/wife*	54 (98.2)	35 (71.4)	21 (52.5)	110 (76.4)
Other family*	43 (78.2)	26 (53.1)	18 (45.0)	87 (60.4)
Employees*	18 (32.7)	27 (55.1)	26 (65.0)	71 (49.3)
Decision regarding cattle made by				
Husband/wife	54 (98.2)	47 (95.9)	39 (97.5)	140 (97.2)
Other family*	22 (40.0)	12 (24.4)	3 (7.5)	37 (25.7)
Employees	0	1 (2.0)	0	1 (0.7)
You, family, employees have contact with other’s cattle*	26 (47.2)	18 (36.7)	29 (72.5)	73 (50.7)
Visitors to cattle				
Wash hands before	6 (10.9)	5 (10.2)	9 (22.5)	20 (13.9)
Clean boots or feet	0	3 (6.1)	2 (5.0)	5 (3.5)
Share equipment with other farmers	20 (36.4)	11 (22.4)	8 (20.0)	39 (27.1)
Farmers in area help each other with cattle	21 (38.2)	21 (42.9)	22 (55.0)	64 (44.4)
New cattle put directly with the herd				
Always	47 (85.5)	37 (75.5)	30 (75.0)	114 (79.2)
Never	2 (3.6)	5 (10.2)	4 (10.0)	11 (7.6)
Sometimes	1 (1.8)	0	1 (2.5)	2 (1.4)
Never bought	3 (5.5)	5 (10.2)	4 (12.5)	13 (9.0)
Missing value	2 (3.6)	2 (4.1)	0	4 (2.8)
Preventive measures for healthy cattle last 12 months				
Tick spray/ dip	54 (98.2)	48 (98.0)	40 (100)	142 (98.6)
Traditional medicine	1 (1.8)	2 (4.1)	0	3 (2.1)
Cattle kept separate from other cattle herds	1 (1.8)	2 (4.1)	4 (10)	7 (4.9)
Antibiotics or other drugs*	39 (70.9)	45 (91.8)	39 (97.5)	123 (85.4)
Deworming	53 (96.4)	46 (93.9)	39 (97.5)	138 (95.8)
Vaccination*	8 (14.5)	2 (4.1)	12 (30.0)	22 (15.2)
Has own bull*	10 (18.2)	24 (49.0)	26 (65.0)	60 (41.7)
Use communal bull*	42 (76.4)	24 (49.0)	14 (35.0)	80 (55.6)
Use artificial insemination	7 (12.7)	1 (2.0)	1 (2.5)	9 (6.3)
Cattle on pasture	51 (92.7)	49 (100)	40 (100)	140 (97.2)
Cattle on pasture tethered*	16 (29.1)	11 (22.4)	1 (2.5)	28 (19.4)
Fenced pastures*				
Yes	24 (43.6)	16 (32.7)	13 (32.5)	53 (36.8)
Yes, partly	19 (34.5)	22 (44.9)	5 (12.5)	46 (31.9)
No	8 (14.5)	11 (22.4)	22 (55)	41 (28.5)
Shared pasture	18 (32.7)	19 (38.8)	24 (60.0)	61 (42.4)
Use pasture in national park*	1 (1.8)	0	17 (42.5)	18 (12.5)
Wildlife on the pasture	22 (40.0)	15 (30.6)	23 (57.5)	60 (41.7)
Water to cattle from open water*	23 (41.8)	9 (18.4)	35 (87.5)	67 (46.5)
Water to cattle from a well*	27 (49.1)	42 (85.7)	2 (5.0)	71 (49.3)
Water to cattle from tap	9 (16.4)	1 (2.0)	4 (10.0)	14 (9.7)
Other livestock on the farm or in the household	52 (94.5)	47 (95.9)	35 (87.5)	134 (93.1)
goat	45 (81.8)	40 (81.6)	28 ()70.0	113 (78.5)
chicken	40 (72.7)	39 (79.6)	28 (70.0)	107 (74.3)
pig*	18 (32.7)	11 (22.4)	2 (5.0)	31 (21.5)
dog*	24 (43.6)	5 (10.2)	8 (20.0)	37 (25.7)
Farmer was satisfied with the health of the cattle over the last 12 months*	43 (78.2)	41 (83.7)	20 (50.0)	104 (72.2)

* Statistically significant (*p* < 0.01) difference between districts at Fisher exact test

Data from an interview with the farm owner or manager of 144 cattle herds in Western Uganda. All variables refer to the last 12 months prior to the sampling occasion. The study was performed in 2015

**Table 2:** Description of some of the numeric parameters regarding cattle herd management and performance in Western Uganda

	Kabarole		Kamwenge		Kasese		Total	
	*n* ^a^	Median^b^ (Q1;Q3)	*n* ^a^	median (Q1;Q3)	*n* ^a^	median (Q1;Q3)	*n* ^a^	median (Q1;Q3)
Calves born*	48	1 (1;3)	46	4 (1.25;6)	39	6 (3;14)	133	3 (1;7)
Abortions*	14	1 (1;1)	12	2 (1;3.25)	18	2 (1.25;3)	44	1.5 (1;3)
Dead cattle*	23	1 (1;1)	26	2 (1;2)	25	3 (1;5)	74	(2 (1;3)
Dead calves	7	1 (1;1.5)	14	2 (1;5.25)	18	2 (1;3.75)	39	2 (1;3)
Sold or given away cattle*	38	1 (1;2)	44	3 (2;4.25)	33	4 (2;6)	115	3 (1.5;4)
Cattle sold for slaughter*	26	1 (1;2)	30	3 (2;4)	30	3 (2;5)	86	2 (1;4)
Cattle sold at market	2	3 (2;4)	18	2.5 (2;3.75)	1	1 (1;1)	21	2 (2;4)
Cattle returned unsold from market	0		2	1.5 (1.25;1.75)	0		2	
Sold or given to another farmer	18	1.5 (1;2)	12	3.5 (1;8.5)	5	3 (3;3)	35	2 (1;3.5)
Cattle sold due to poor performance	5	1 (1;2)	9	2 (1;3)	11	2 (1.5;3.5)	25	2 (1;3)
Cattle sold due to disease	3	1 (1;1)	4	1 (1;1.25)	5	4 (1;5)	12	1 (1;2.5)
Cattle bought or received	15	2 (1;2)	32	2 (1;4)	18	2 (1;3)	65	2 (1;3)
Cattle bought from market	2	1 (1;1)	12	1 (1;2)	4	1.5 (1;2.25)	18	1 (1;2)
Cattle bought/received from other farmer	13	2 (1;2)	22	2.5 (1;4.74)	14	2 (1;2.75)	49	2 (1;4)
Cattle bought with known disease	0	–	6	1 (1;1)	1	1 (1;1)	7	1 (1;1)

*Statistically significant (*p* < 0.01) difference between districts at Kruskal Wallis test

^a^Number of responses with value >0, included in the median value

^b^Median value based on responses >0

Data from an interview with the farm owner or manager. All variables refer to the last 12 months prior to the sampling occasion. The study included 144 farms and was performed in Western Uganda in 2015

The majority of farmers had sold or given away cattle the last year, mainly for slaughter. According to the local field teams there is a weekly livestock market in Kamwenge but no such market in Kabarole nor in Kasese, except for one on the border to the Democratic Republic of Congo where cattle transported from Tanzania are also traded. However, it was noted that the weekly markets often had a section for livestock, poultry and meat. The farmer interviews gave the same picture where 18 famers in Kamwenge had sold cattle at a market but only two in Kabarole and one in Kasese.

In addition, almost half of the farmers had bought or received new cattle, mainly from other farmers and less frequently from a livestock market. The majority (79.2%, *n* = 114) said that they always put new animals directly together with the rest of the herd. Of the 11 farmers that used some type of quarantine for new cattle, a separate pasture or paddock or at another home was stated as the quarantine location. Seven farmers (one in Kabarole, two in Kamwenge and four in Kasese) said they kept their cattle separated from other cattle herds (Table 1). Biosecurity measures like visitors’ hand-wash or cleaning of boots or feet were rarely practiced.

Overall, almost three quarters of farmers were satisfied with the health status of their cattle the preceding 12 months, however in Kasese this figure was only 50%. In addition, 123 (85%) stated that they had given antibiotics or other drugs as prevention to healthy cattle and 142 (99%) had used tick spray during the same time period (Table 1). Of the farmers that stated they had had their cattle vaccinated (*n* = 22), the most frequent diseases stated as vaccinated against were FMD (*n* = 10), blackleg (*n* = 5), ECF (*n* = 2) and one each for brucella, anaplasma and lumpy skin disease. The time of vaccination was often not clear and could be up to “several years ago”.

The types of wildlife some farmers (41.7%, *n* = 60) stated as present in their pastureland were mainly monkeys and baboons (*n* = 26) and a wide range of animals mentioned by one or a few farmers including wildcats, foxes, elephants, buffalos, wild pigs, mongoose, antelopes, hyenas, rabbits, lions, hippos, and crocodiles or “animals from Queen Elizabeth National Park”.

Water sources differed somewhat between the three districts. In Kabarole 16.4% (*n* = 9) of the farmers stated that they gave the animals water from a tap, while 49.1% (*n* = 27) said they took water from a well and 41.8% (*n* = 23) that the cattle drank from an open water source. In Kamwenge, the corresponding figures were 2.0% (*n* = 1) tap water, 85.7% (*n* = 42) from a well and 18.4% (*n* = 9) from an open source while in Kasese they were 10.0% (n = 4) tap water, 5.0% (*n* = 2) from a well and 87.5% (*n* = 35) from an open water source.

### Serological results

Serum was collected from in total 1567 individual cattle; 901 young and 666 adults. The median number (1st; 3rd quartile) of individuals per herd was 8 (4;20). The median number (1st; 3rd quartile) of analysed samples per herd was 7 (4;12), 7 (4;16) and 7 (4;7) for brucella, salmonella and BVD, respectively.

The apparent herd prevalences of the three diseases are presented in Table 3. The difference between districts was statistically significant (*p* < 0.001) for brucella but not for salmonella and BVD (*p* > 0.05). Kasese had the highest apparent herd prevalence of brucella and Kabarole the lowest. Approximately half of the herds in all three districts were positive for BVD. The apparent herd prevalence when test results from young or adult individuals were assessed separately are shown in Table 4. There was a trend that herds in Kasese tested positive to a higher number of diseases than herds in Kamwenge. Herds in Kabarole were to a larger extent negative or only positive to one disease (Table 5). For BVD, 48 of 138 herds had at least one positive young individual and of those, 24 had two or more positive young individuals, suggesting an ongoing infection in the herd.

**Table 3:** Frequency and apparent prevalence (%) of seropositive cattle herds in Western Uganda

	Kabarole *n* (%)	Kamwenge *n* (%)	Kasese *n* (%)	Total *n* (%)^a^
Total	55	49	40	144
Brucella^b^	3 (5.5)	5 (10.2)	16 (40)	24 (16.7 (11.0;23.8))
Salmonella	8 (14.5)	16 (32.7)	10 (25.0)	34 (23.6 (16.9;31.4))
BVD	28 (50.9)	27 (55.1)	22 (55.0)	77 (53.4 (45.0;61.8))

^a^With exact binomial confidence intervals of %

^b^Significant difference (*p* < 0.001) between districts with two-sided Fisher’s exact test

**Table 4:** Frequency and prevalence (%) of seropositive cattle herds based on tested young or adult individuals

	Herds with young^a^ individuals		Herds with adult^a^ individuals	
	n positive herds (n tested)	prevalence positive herds (%)^b^	n positive herds (n tested)	prevalence positive herds (%)^b^
Brucella	4 (123)	3.3 (0.9;8.1)	21 (137)	15.3 (9.7;22.5)
Salmonella	15 (138)	10.9 (6.2;17.3)	20 (129)	15.5 (9.7;22.9)
BVD	48 (138)	34.8 (26.9;43.4)	40 (89)	44.9 (34.4;55.9)

^a^young <2 years, adult >2 years

^b^With exact binomial confidence intervals

Prevalence of herds that were sero-positive to brucella, salmonella, and BVDV. A herd was classified as positive if at least one sampled individual animal tested positive in the respective ELISA, using the manufacturers’ cut-offs. The number of sampled animals per herd was; all for herds with up to 20 cattle, 20 for herds with 21 to 50 cattle, and 30 if there were more than 50 cattle. When feasible, individuals up to 2 years of age were sampled. First, samples from seven individuals were tested serologically prioritising young individuals for salmonella and BVD, and older for brucella. If all were negative, for brucella the remaining samples from the herd were analysed. For salmonella another nine samples were analysed, if the herd was still negative any remaining samples were analysed. The study was performed in Western Uganda in 2015

**Table 5:** Number of diseases cattle herds were positive to

Number of ELISA tests for which the herd had a positive test result *	Kabarole *n* (%)	Kamwenge *n* (%)	Kasese *n* (%)	Total *n* (%)
0	22 (40.0)	13 (26.5)	11 (27.5)	46 (31.9)
1	28 (50.9)	26 (53.1)	12 (30.0)	66 (45.8)
2	4 (7.3)	8 (16.3)	15 (37.5)	27 (18.8)
3	1 (1.8)	2 (4.1)	2 (5.0)	5 (3.5)

* Difference between districts tested with two-sided Fisher’s exact test; *p* < 0.007

Frequency and prevalence of herds that were sero-positive (at least one individual with a positive test result) to none, one or several of brucella, salmonella or BVD in each study district. The study was performed in cattle herds in Western Uganda in 2015

### Associations between herd management and biosecurity and infectious disease burden

The poisson model suggested that having employees to care for the cattle was associated with a herd being positive to a higher number of diseases with an RR estimate (95% CI) of 1.9 (1.3;2.8). In addition, an increasing number of dead cattle the previous 12 months and sharing pasture both had RR estimates (95% CI) of 1.1 (1.0;1.1).

**Fig. 2:**
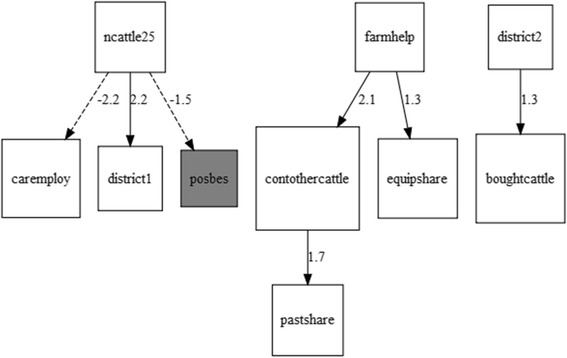
Final global Directed Acyclic Graph (DAG) from additive Bayesian network modelling. The DAG illustrates direct and indirect associations between herd status (positive/negative serology) to at least one of brucella, salmonella or BVD, and farmer interview answers relating to herd biosecurity. A heuristic search with 1000 iterations was run with up to 2 parents allowed. Arcs that were included in at least 50% of local models are shown. Variables with no arcs are not shown. The numbers on the arcs represent the posterior marginal densities; a dashed line indicate negative association. All variables were binomial (no/yes). Variable coding: posbes = herd sero-positive to at least one of the diseases, district1 = Kabarole, district2 = Kamwenge, ncattle25 = herd size 2-5 cattle, careemploy = employees care for cattle, pastshare = share pasture with other cattle herds, boughtcattle = bought or received cattle the last 12 months, equipshare = share equipment with other farms, contothecattle = people from the farm has contact with cattle form other farms, farmhelp = farmers in the area help each other with their cattle

The globally best fitting DAG from the multivariate model is presented in Fig. 2. It included ten nodes and seven arcs in three separate networks. Variables for quarantine for new cattle, having visitors in contact with the cattle wash hands or boots, and the categories of herd sizes >5 were not connected to any other nodes and are not shown. The global fit of the model did not increase beyond 1 parent (network score = −1258). The first network illustrates how small farms (2–5 cattle) were less likely to have employees care for their cattle, or to be positive to at least one disease, as well as more likely to be from district 1 (Kabarole). The second network of variables illustrates how farmers who help each other were more likely to also share equipment, the cattle to have contact with other cattle and to share pasture. The third network illustrates how farms in district 2 (Kamwenge) were more likely to have bought cattle. The second and third networks illustrate how herd parameters are associated with each other, although not to herd serological status.

## Discussion

### Potential for improved herd biosecurity

A shared characteristic of the three model diseases is that they can be controlled and prevented by biosecurity measures. Zoonotic infections such as salmonellosis and brucellosis are particularly important to control in countries such as Uganda with a large rural population with close contact with livestock, and dairy and meat value chains that may lack the capacity to control these pathogens [39, 40]. Studies on herd biosecurity routines and basic production aspects in Ugandan cattle farms are sparse. The results from the farmer interviews indicate that there is a huge potential for improved biosecurity. For example, the proportion of farmers that had introduced new cattle in the last 12 months was high while very few stated that they used a quarantine for new cattle. Given that most farmers had received only a few animals, it should be possible to keep them isolated for a limited time period. Most cattle that were sold were destined for slaughter while most cattle that were brought in came from another farmer, and only a few from the cattle market. Cattle markets are relatively rare in the study area but the finding is, nevertheless, positive from a disease control point of view. The proportion of farmers who had traded diseased cattle was low, which is also positive from a disease control perspective. This figure may however be an under-estimation due to reluctance to divulge such habits if it was perceived to be regarded as poor practice by the researchers.

In Kabarole district, land ownership allows more farmers to have paddocked pasture for their cattle. This should reduce the risk of disease transmission because the herd is separated from other herds. For farmers that use communal grazing, a disease prevention measure could be for the community to treat their cattle as one epidemiological unit and to keep their common herd separate from the herds of other communities. A well or open water were the most common sources of water, and watering points are sites where herds mix. Agreements could be made between communities to visit the watering point at different times, to separate herds in space and time and thus reduce the risk of direct disease transmission.

Biosecurity routines, such as handwashing and cleaning boots for visitors to the cattle, were rarely used. Many basic measures such as an extra pair of boots for visitors, asking visitors to wash their hands before handling cattle, a small paddock to keep new or diseased cattle isolated, and to only buy healthy looking cattle are all relatively low-cost investments which, if they were practiced, would reduce the risk of introduction of infectious diseases to the herd. Advice on preventive actions is difficult to convey, since what is sought is a non-event (absence of disease or production loss) and hence the incentives are not tangible. This is a particular challenge in settings where farmers might not feel empowered or in charge of their own destiny and where uncontrollable disasters and political unrest are recurrent. In addition, biosecurity measures have to be practically feasible for farmers to adopt them, and knowledge about what the local population of farmers can actually do, or think they can’t do, is needed. This will be the focus of some of our future studies.

Farmer knowledge of infectious diseases and biosecurity was not directly assessed. Surveys from other low-income countries with small-scale farming have suggested a general lack of knowledge and access to information on infectious diseases and control measures. Studies in Uganda have found that small-holders with pigs had knowledge about African swine fever but did, however, not implement biosecurity [14, 41].

The risk factor analysis suggested an association between the number of diseases a herd was serologically positive for and the following factors: employees caring for the cattle, shared pasture and a higher number of dead cattle. While this association does not necessarily reflect a causation, the first two could be explained as related to indirect and direct contacts with other herds, while the third is most likely an indication of the presence of disease in the herd and hence a “risk factor” that is in fact a symptom. However, risk factors are often correlated and individual effects may be difficult to discern. The ABN is one approach to tackle this challenge. The results from the ABN suggested that a herd size of two to five cattle was associated with a herd being negative to all three diseases, and not having employees caring for the cattle. The DAG from the ABN illustrates connections between variables that the univariate analysis did not capture. This method could potentially be used to create “farmer profiles” in the study population and suggest how, within a small geographical area, farmers vary in their management. For biosecurity interventions it would be very helpful to know which practices are likely “to come in a package” and thus need to be addressed as such. Moreover, it is important to know if farmers should be addressed as a community rather than individual farmers because their cattle are kept as one epidemiological unit. This was supported by the ABN results where a cluster with shared pasture, shared equipment and farms helping each other emerged. A larger sample size might had given enough power to detect more risk factors in the poisson regression model and to generate more complex DAGs.

During the field work it was noted how heterogeneous the farmer population is in the study area. Not only due to parameters that were assessed in the interviews, but to factors related to cultural group, socio-economic status, and experience as cattle owners. This could complicate the assessment of differences between farms with healthy and less heathy cattle, i.e. make identification of single risk factors for disease harder to identify. The three districts differed in some aspects, where a few could be seen in the DAGs (Fig. 2), e.g. Kabarole having smaller farms managed by the farmer and his/her family. There were some characteristics that were different for the farms in Kasese when compared to those in Kabarole and Kamwenge. The herds were larger and used more open water sources and pasture in natural parks or game reserves. They appeared to be more likely to be managed by the husband/wife of the family with less involvement of other relatives, and employees taking care of the cattle. They sold more cattle and the farmers and their employees were more frequently in contact with other cattle. Still, the overall heterogeneity of the farms and farmers made subgroups less distinguishable by the current study design.

### Serological results

The results indicate that the three model diseases are endemic at a moderate (brucella and salmonella) to high (BVD) prevalence in the Ugandan cattle population. The diseases were chosen because they have different epidemiology and transmission routes and affect herd productivity.

The prevalence of salmonella in animal production is high in many parts of the world. Production systems that cause continuous exposure to salmonella are usually intensive [42, 43] but in more extensive systems the animals may be exposed to environmental bacteria, for example where they are gathered at night or in the milking kraal. The prevalence of salmonella isolated from mesenteric lymph nodes from healthy cattle slaughtered in an abattoir in north-east Nigeria was 61% [44], although the authors remark that this was probably an over-estimation because of the stress animals had been subjected to before being culled. Nevertheless, salmonella can be expected to circulate in smallholder farming systems in Africa, which the current results support, and measures to prevent exposure of the animals to salmonella from other herds and/or the environment would be expected to reduce the prevalence.

BVD has not been extensively investigated in smallholder systems in Africa. A study among small-scale family farms in northern Turkey revealed a seroprevalence of 32% [45]. In Kerala province in India, a study among smallholder dairy cattle revealed a prevalence of 24.7%, but higher among animals with infertility problems [46]. There are some reports of BVD being endemic in African cattle, from South Africa [47] and Cameroon [26]. A Swedish MSc thesis detected serological reactions among cattle in peri-urban farming systems in Kampala [25]. In addition, serological reactions to BVDV have been detected in African wildlife such as kudu and eland [48]. BVD has an immunosuppressive effect and in populations with a high infection pressure, e.g. sub-Saharan Africa, this impact on health and performance is likely to be even more important. Reducing the high seroprevalence found in this study might therefore have a positive effect in cattle herds in Uganda.

There are various reports on the presence of brucellosis in African cattle. In Uganda, serosurveys show different herd prevalence levels ranging between 5 and 100% in different regions, agro-ecological zones, and different animal management systems [21, 22, 24, 49] and it is not always clear to what extent vaccination was practised in the study populations. No reports on prevalence from the particular area of this study have been found but it was clear that brucellosis is endemic.

There was a difference in prevalence between districts for Brucella as well as for the number of diseases herds were positive to, both had the highest numbers in Kasese district. Sharing pasture remained in the final poisson model of the outcome number of diseases a herd was positive to. Kasese had many farms with communal grazing, however district did not remain in the model. The ABN suggested that herds that were negative to all three diseases, were linked to fewer employees, small herd size, and being located in Kabarole. Brucella has been shown to be more prevalent in pastoralist communities with communal grazing, both in livestock and in humans [18], agreeing with the current results.

Interestingly, a clear majority of all farmers (72%) were satisfied with the health status of their cattle the last year, although this figure was lower for the farmers in Kasese. This supports that a high burden of clinical and subclinical infections with negative effects on productivity might be considered as normal in the study population. Similarly, abortions could be part of what is experienced as “normal”, although abortions were more frequent in Kasese. Abortions were likely under-reported by farmers because pregnancy checks are hardly ever performed and abortions in early stages of pregnancy would be noted as a prolonged calving interval. This, in turn, might not be noted either, because there were generally no farm records. In a previous study, Kenyan farmers have been suggested to report too low numbers of abortions in their cattle [50].

### Study limitations and practical challenges with impact on methodology and results

There are challenges to carrying out epidemiological field studies in low-income countries. Failure to acknowledge these challenges may lead to invalid results being presented as solid data.

Firstly, in the absence of formal or informal animal registers, any sampling frame will be incomplete and the level of representativity is often difficult to assess. Even when a lot of effort is put into applying a probability sampling method, practical obstacles such as inaccessibility due to poor roads and failure to communicate regarding visiting time often lead to replacement of selected farms in a manner that sometimes impact on the sampling method. In the present study, the aim was a random sample of villages, which for practical reasons was not fully achieved. However, the team members that recruited farmers did at that point not know which factors that would be evaluated, and the first author had no knowledge about the villages in the area. This would have avoided any subconscious selection bias. Two herds per village were sampled which, in theory, would call for village to be included as a random variable in models. However, village is not an epidemiological unit in Uganda but an administrative and geographical delimitation of the rural landscape where farms are continuously scattered and usually not aggregated near a village centre. Hence, no actual clustering on village level is to be expected.

Secondly, cultural clashes and language barriers may lead to misunderstandings that affect the results of interviews and questionnaires. The extent of this is difficult to assess. Including control questions to detect bias or misunderstandings may lead to further complications and distrust. One factor not to be ignored in this type of studies is the power imbalance and skewed relation when European, well-educated researchers perform field studies in farms in low-income countries. It is well-known that interview subjects might say what they think the interviewer (or researcher present in the interview situation) wants to hear, in favour of the most truthful answer. One should have in mind that highly unequal relationships such as in this study could increase the response bias. Efforts were made at each interview to inform the farmer that their name would not be published, who the team members were and what organisation they represented for the study. Still, it cannot be known whether the farmers felt confident enough to supply truthful answers to all questions.

The aim of 60 sampled herds per district was not reached, mainly in Kamwenge and Kasese. This was because of the unexpectedly large amount of time needed for transportation between farms, time spent at each farm visit, and farmers that had forgotten about the agreed visit and/or had already let their cattle out on pasture when the team arrived. Because of budget and time limitations, the field work could not be extended until all 180 farms had been visited. Efforts were made to cover as many geographical areas as possible during sampling. Only one farmer declined to participate in the study at the first recruitment visit. The authors believe the results to fairly represent the situation of bovine infectious diseases and cattle management in the study area, as well as other regions in Uganda with similar conditions.

Serology does not indicate the current infection status but rather the infection history of the herd. By prioritising individuals of less than 2 years’ age for salmonella and BVD a more recent history could be assessed. Nevertheless, a cross-sectional study is a snapshot of serological status and a longitudinal study design might have given more information about infection dynamics in the population. It is not known if there are seasonal variations in three model diseases in the study area, a fact that would clearly affect the results. However, cattle production itself does not appear to change by season in the study area so there is no obvious reason for seasonality in the disease prevalence.

The herd sensitivity is lower for farms with few tested individuals and hence the risk of false negatives increases. However, a cut-off of one positive individual per herd reduces herd specificity, especially with the sample analysis approach used in this study. Increasing the cut-off for a herd to be classified as positive to two seropositive individuals (of any age) changed the number of positive herds from 24 to 12 for brucella, from 34 to 6 for salmonella, and from 77 to 37 for BVD. This reduction of >50%, highlights the importance of case definition for prevalence estimates. In addition, changing the cut-off could influence a risk factor analysis. For BVD, at least three positive samples from individuals has been used as a cut-off for the herd to be likely to have persistently infected (PI) animals, i.e. an ongoing infection, as herds without PI animals tend to self-clear from the infection [51]. However, in the current study the objective was not to identify herds with PI animals but herds with animals that were seropositive, i.e. had undergone the infection.

The ELISAs used for the serological analyses were not evaluated in the study population. It is known that tests validated in non-exposed populations perform differently in endemic situations and this could have affected the results in the current study [52]. However, there are no available commercial tests for the diseases in this study that are properly evaluated in African settings and perhaps no market incentive to perform such evaluations of the current tests. The salmonella ELISA does not detect all serotypes and hence the real seroprevalence of salmonella is likely to be higher.

## Conclusions

There is room for improvement of biosecurity practices in Ugandan cattle production. However, to take proper action to improve biosecurity and cattle health in any region, knowledge of the current status is necessary, i.e. disease prevalence and a description of management systems. Design and evaluation of interventions for such improvement would benefit from the creation of farmer profiles, using data on disease prevalence and herd management practices. As in many studies on biosecurity, few significant associations were seen in the risk factor analysis but this does not justify lack of efforts to improve biosecurity.

Despite the challenges and limits of this study, it is clear that salmonella, brucella and BVD are present in cattle in the study area. Moreover, the prevalence of these infections is, to some extent, associated with farm management practices. Brucellosis and salmonellosis are important zoonotic infections and reducing the prevalence within and between herds would benefit the human population directly as well as allow for healthier livestock.

## Additional file

Additional file 1: The questionnaire used for the farmer interviews. (PDF 281 kb)

## Abbreviations

AI: Artificial insemination
BVD: Bovine viral diarrhoea
DAG: Directed acyclic graph
DVO: District veterinary officer
ECF: East Coast fever
FMD: Foot-and-mouth disease
TAD: Transboundary animal disease
