# Supplementary material for: Biosecurity aspects of cattle production in Western Uganda, and associations with seroprevalence of brucellosis, salmonellosis and bovine viral diarrhoea
Source: BMC Vet Res. 2017 Dec 6;13:382. doi: 10.1186/s12917-017-1306-y (PMC5719755; doi:10.1186/s12917-017-1306-y)
Supplement: Additional file 1: — The questionnaire used for the farmer interviews. (PDF 281 kb) [file 12917_2017_1306_MOESM1_ESM.pdf]

Date Name of person who did the interview _______________

Farmer questionnaire

Name of farmer Ms/Mr _____________________________ Mobile phone nr ______________

District ________________ Sub-county ________________ Village ________________

Cattle are kept for (circle) Dairy Beef Dairy and beef

Breeds of cattle? Indicate with 1 = main breed, 2 = second common, 3 = least common, 4 = none:

Local breeds ______ Mix with exotic ______ Exotic ______

How many cattle do you have (circle)? 2-5 5-10 10-20 20-50

50-100 100-150 150-200 > 200

How many calves were born the last 12 months? ___________

How many cows did have an abortion the last 12 months? ___________

How many cattle died the last 12 months? ___________

Of dead cattle, how many were calves < 1 year old? ___________

How many cattle in total did you **sell or give away** the last 12 months? ___________

Of those, how many cattle did you sell for slaughter? ___________

How many cattle were sold on the market? ___________

How many were taken to the market but returned to the farm? ___________

How many were sold directly to another farmer? ___________

How many cattle were sold because they did not perform well? ___________

How many cattle were sold because they were diseased? ___________

How many cattle in total did you **buy or receive** the last 12 months? ___________

Of those cattle - how many were bought from the cattle market? ___________

- how many were bought directly from another farmer? ___________

- how many were diseased when you bought them? ___________

Do you put new cattle in your herd directly? Always Sometimes Never

If not, where do you keep new cattle? ___________________________________

For how long are they kept separate? ___________________________________

Who takes care of cattle? Wife/ Husband Other family/ relatives Employees

Who makes decisions regarding cattle? Wife/ Husband Other family/ relatives Employees

Do you, your family or employees have contact with other cattle (not yours)? Yes No

Do visitors like neighbours, NAADS, advisors wash hands before contact with cattle? Yes No

Do visitors change or clean their boots/ feet before contact with your cattle? Yes No

Do you share crush or other equipment with other cattle farmers? Yes No

What task involving cattle handling do cattle farmers from your area help each other with?

None Yes, please describe ____

What did you do to **prevent healthy cattle from getting diseased** the last 12 months?

Tick spray/dip Gave traditional medicine Kept cattle separate from other cattle

Gave antibiotics or other medicine Deworming Vaccination, to what? __________________

Do you use AI and/or bull (circle)? AI Own bull Local village/communal bull

If own bull is used, how often/ when do you buy a new bull? ________________________

Do you keep your cattle on pasture? Yes Yes, tethered No

Are pastures fenced? Yes Partly No

Do you share pasture with other herds so the cattle mix? Yes No

Do you use pasture in National Parks or protected area? Yes No

Do wild animals come to your environments? Yes, name species _________________ No

How are cattle watered? In open water (e.g. a river) Water from a well Tap water

Do you have other livestock than cattle? Yes, name species ________________________ No

Are **you satisfied** with the health status of your cattle over the last 12 months? Yes No
